# Inhalational Injury Secondary to House Fire

**DOI:** 10.21980/J8TW7N

**Published:** 2023-10-31

**Authors:** Ryan O’Neill, Benjamin M Ostro, Jennifer Yee

**Affiliations:** * The Ohio State University, Department of Emergency Medicine, Columbus, OH

## Abstract

**Audience:**

This scenario was developed to educate emergency medicine residents on the diagnosis and management of patients with an inhalational airway injury secondary to a house fire.

**Background:**

Burn injuries are a common occurrence encountered by the emergency physician. According to the National Hospital Ambulatory Medical Care Survey, around 371,000 patients were treated in emergency departments for fire or burn injuries across the United States in 2020. This represents around 1% of emergency department visits related to injury, poisoning, or adverse effects.^1^ One of the most dangerous and time critical aspects of managing severely burned patients is inhalation injury. Inhalation injury is a relatively vague term which may refer to pulmonary exposure to a wide range of chemicals in various forms. In the context of burn patients, this is most often smoke exposure. It is critical that the emergency medicine provider rapidly identifies the potential for an inhalational injury in order to determine the need for definitive airway management. It is also important that the provider has the necessary skills and systematic approach to manage what is likely to be a difficult airway. Furthermore, providers must then have the knowledge of how to best manage and resuscitate these severely burned patients post-intubation.

**Educational Objectives:**

At the conclusion of the simulation session, learners will be able to: 1) recognize the indications for intubation in a thermal burn/inhalation injury patient; 2) develop a systematic approach to an inhalational injury airway; and 3) recognize indications for transfer to burn center.

**Educational Methods:**

This session was conducted using high-fidelity simulation, followed by a debriefing session and lecture on the diagnosis, differential diagnosis, and management of inhalational airway injury secondary to a house fire. Debriefing methods may be left to the discretion of participants, but the authors have utilized advocacy-inquiry techniques. This scenario may also be run as an oral board case.

**Research Methods:**

Our residents are provided a survey at the completion of the debriefing session so they may rate different aspects of the simulation, as well as provide qualitative feedback on the scenario. The local institution’s simulation center’s electronic feedback form is based on the Center of Medical Simulation’s Debriefing Assessment for Simulation in Healthcare (DASH) Student Version Short Form^2^ with the inclusion of required qualitative feedback if an element was scored less than a 6 or 7.

**Results:**

Nine learners completed a feedback form. This session received all 6 & 7 scores (consistently effective/very good and extremely effective/outstanding, respectively) other than one isolated 5 score.

**Discussion:**

This is a cost-effective method for reviewing inhalational airway injury diagnosis and management. The case may be modified for targeted audiences, expected resources, and learning objectives, such as removal of a bronchoscope availability in settings which are expected to be resource-limited. Some readers may choose to focus on other aspects of burn management instead of airway securement such as cyanide and/or carbon monoxide toxicity. We encourage readers to limit the number of learning objectives because airway algorithms and troubleshooting for this scenario was a rich, stand-alone debriefing. There was not enough time to review in detail all nuanced aspects of the burned patient, including: Lund-Browder versus rule of 9’s, modified Brooke versus Parkland formulas, indications for and completion of escharotomies, and/or identification and treatment of cyanide and carbon monoxide toxicity.

**Topics:**

Medical simulation, burns, airway emergencies, emergency medicine.

## USER GUIDE

[Table t1-jetem-8-4-s49]

**Table t1-jetem-8-4-s49:** 

List of Resources:	
Abstract	49
User Guide	51
Instructor Materials	53
Operator Materials	67
Debriefing and Evaluation Pearls	69
Simulation Assessment	75

**Learner Audience:**

Interns, Junior Residents, Senior Residents

**Time Required for Implementation:**

**Instructor Preparation:** 30 minutes

**Time for case:** 20 minutes

**Time for debriefing:** 40 minutes

**Recommended Number of Learners per Instructor:**

3–4

**Topics:**

Medical simulation, burns, airway emergencies, emergency medicine.

**Objectives:**

By the end of this simulation, the learner will be able to:

1. Recognize the indications for intubation in a thermal burn/inhalation injury patient
2. Develop a systematic approach to inhalational injury
3. Recognize indications for transfer to burn center

### Linked objectives and methods

Patients with inhalation injury require rapid assessment and prognostication of their airway. After this scenario, providers will be able to recognize the indications for intubation in a thermal burn/inhalation injury patient (Objective 1). Inhalation injuries often present with difficult airways secondary to edema and obscured anatomic landmarks. This scenario will give providers an opportunity to develop and practice a systematic approach to the difficult airway (Objective 2). Finally, providers will learn how to recognize indications for transferring patients to a burn center (Objective 3). Objectives were tracked by facilitators taking notes during the simulation scenario for the subsequent debriefing discussion. This simulation scenario allows learners to reinforce inhalational injury management skills in a psychologically-safe learning environment, and then receive formative feedback on their performance.

### Results and tips for successful implementation

This simulation was written to be performed as a high-fidelity simulation scenario, but also may be used as a mock oral board case.

The case was written for emergency medicine residents within the setting of an ED. However, surgery and anesthesia will be unavailable for consultation if called. The setting may occur in a burn center or in a more rural setting requiring ultimate transfer, depending on the vision of the facilitator.

Starting the case with an oxygen saturation of 90% while on a non-breather mask and with the patient able to speak in one to two word sentences (although with stridor) led some learners to believe that the airway was not an imminent concern. Depending on the experience of the learners, these factors may be edited at the facilitator’s discretion.

Our scenario began with Emergency Medical Services (EMS) emphasizing that the patient was found unconscious on the couch without signs of trauma or falling debris in the house. This allowed for residents to attribute post-intubation hypotension as possibly secondary to cyanide toxicity rather than hemorrhagic shock. Multiple groups paralyzed with rocuronium because they did not identify concern for a difficult airway which may benefit from an “awake look.” Several also voiced concern for use of succinylcholine in a patient with burns, even though the burn occurred acutely.

One of our clinical respiratory therapists participated in the case to help assist with equipment set-up, tube securement, and bagging. We used both a whole-body manikin to start the case, as well as a burn victim airway task trainer for nasopharyngeal/oropharyngeal management. Once the team initially voiced that they wanted to secure the airway, the respiratory therapist moved them to a side table with the burned airway task trainer with a non-rebreather mask in place, which had been previously covered with a sheet. The team was able to perform direct laryngoscopy, video laryngoscopy, and bronchoscopy on the burned airway task trainer. If the residents moved towards cricothyrotomy, they were directed back to the whole-body manikin which had this capability. Facilitators who do not have a burned airway task trainer may choose to use a full-body manikin for all their airway steps, voicing out loud that there is too much edema to safely pass a large endotracheal tube and/or too much soot to clearly see the vocal cords.

Nine learners completed a feedback form. This session received all 6 and 7 scores (consistently effective/very good and extremely effective/outstanding, respectively) other than one isolated 5 score. The lowest average score was 6.55 for “Before the simulation, the instructor set the stage for an engaging learning experience.” The highest average score was 7 for “The instructor structured the debriefing in an organized way.” The form also includes an area for general feedback about the case at the end. Illustrative examples of feedback include: “Great case & great learning points.” Specific scores are available upon request.

## Supplementary Information



## Figures and Tables

**Table t2-jetem-8-4-s49:** 

#1	Electrocardiogram, sinus tachycardia
#2	Chest X-ray
#3	Chest X-ray, post endotracheal intubation
#4	Complete blood count (CBC)
#5	Basic metabolic panel (BMP)
#6	Hepatic function panel
#7	Creatine kinase
#8	Lactate
#9	Prothrombin Time (PT)/International Normalized Ratio (INR)
#10	Troponin
#11	Ethanol level
#12	Toxicology Screen
#13	Arterial blood gas
#14	Arterial blood gas (post-intubation)
#15	Co-oximetry

**Table t3-jetem-8-4-s49:** 

**Vitals:**	Heart Rate (HR) 120	Blood Pressure (BP) 140/96
	Respiratory Rate (RR) 24	Temperature (T) 99.8°F
	Oxygen Saturation (O_2_Sat) 90% on 15-liter non-rebreather mask	

**Figure f1-jetem-8-4-s49:**
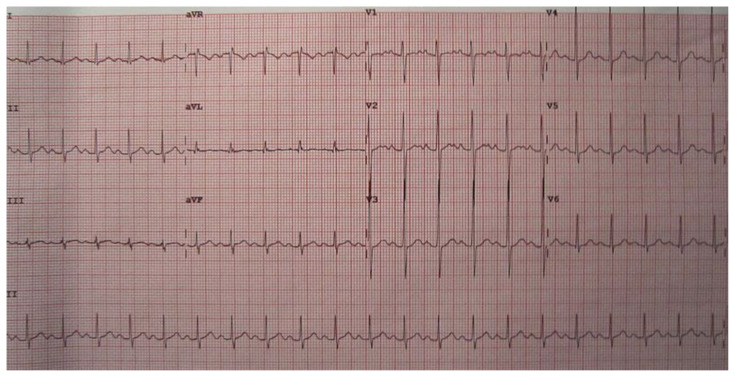


**Figure f2-jetem-8-4-s49:**
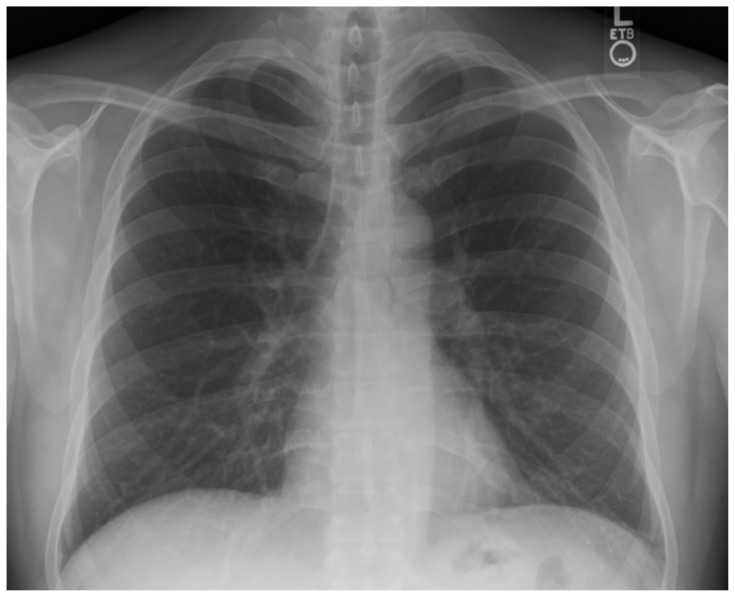


**Figure f3-jetem-8-4-s49:**
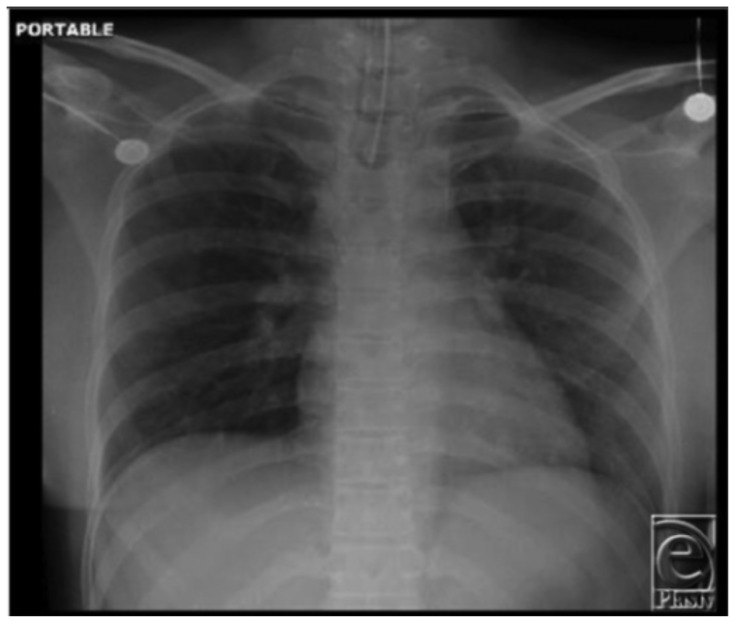


**Table t4-jetem-8-4-s49:** 

*Complete blood count (CBC)*	
White blood count (WBC)	16.4 × 1000/mm^3^
Hemoglobin (Hgb)	15.0 g/dL
Hematocrit (HCT)	48%
Platelet (Plt)	580 × 1000/mm^3^

**Table t5-jetem-8-4-s49:** 

*Basic metabolic panel (BMP)*	
Sodium	138 mEq/L
Potassium	4.0 mEq/L
Chloride	98 mEq/L
Bicarbonate (HCO_3_)	20 mEq/L
Blood Urea Nitrogen (BUN)	30 mg/dL
Creatinine (Cr)	1.0 mg/dL
Glucose	90 mg/dL
Calcium	8.0 mg/dL

**Table t6-jetem-8-4-s49:** 

*Liver function test (LFTs)*	
Total bilirubin	0.8 mg/dL
Direct bilirubin	0.2 mg/dL
Alkaline phosphatase	100 units/L
Aspartate aminotransferase (AST)	30 units/L
Alanine aminotransferase (ALT)	40 units/L
Albumin	3.0 g/dL

**Table t7-jetem-8-4-s49:** 

*Creatine kinase*	40 U/L

**Table t8-jetem-8-4-s49:** 

*Lactate*	8.2 mmol/L (<1.0 mmol/L)

**Table t9-jetem-8-4-s49:** 

*Coagulation Studies*	
Prothrombin time	11 seconds
International normalized ratio	0.8

**Table t10-jetem-8-4-s49:** 

*Troponin*	<0.015 ng/mL

**Table t11-jetem-8-4-s49:** 

*Ethanol*	< 0.01 mg/mL

**Table t12-jetem-8-4-s49:** 

*Toxicology Screen*	
Amphetamines	Negative
Barbiturates	Negative
Benzodiazepines	Negative
Cocaine	Negative
Methadone	Negative
Opiates	Positive
Oxycodone	Negative
PCP	Negative
THC	Negative

**Table t13-jetem-8-4-s49:** 

*Arterial blood gas*	
pH	7.35
pCO_2_	45 mmHg
pO_2_	57 mmHg
HCO_3_	20 mEq/L
O_2_ Saturation	90% on a non-rebreather

**Table t14-jetem-8-4-s49:** 

*Arterial blood gas, post-intubation*	
pH	7.33
pCO_2_	50 mmHg
pO_2_	400 mmHg
HCO_3_	20 mEq/L
O_2_ Saturation	94% on FiO2 100%

**Table t15-jetem-8-4-s49:** 

*Co-oximetry*	
pO2	400
COHgb	12

**Table t16-jetem-8-4-s49:** SIMULATION EVENTS TABLE:

Minute (state)	Participant action/ trigger	Patient status (simulator response) & operator prompts	Monitor display (vital signs)
0:00 (Baseline)	SP nurse brings the team into the patient’s room in the emergency department.	Participants should begin by placing the patient on a monitor, obtaining a focused history from EMS and patient, and performing a primary physical exam.	A: T 99.8°F HR 120 BP 140/96 RR 24 O_2_ sat 90% on a non-rebreather mask
2:00	Team should recognize respiratory distress and impending need for intubation. IV placed, labs, EKG should be ordered.	If team vocalizes need for intubation, the respiratory therapist will direct them to a side table with the burned airway task trainer previously covered with a sheet. The team is expected to tell the respiratory therapist their plan for airway management without prompting. If anesthesia and/or surgery are called, they are currently unavailable and will call back when they are free.	A: T 99.8°F HR 120 BP 140/96 RR 24 O_2_ sat 90% on a non-rebreather mask
3:00	Team should recognize worsening hypoxia during prolonged intubation attempts if sedated with any meds other than ketamine. Team should ventilate the patient with the bag-valve mask in between attempts and move decisively down a pre-determined airway pathway with different techniques rather than trying the same method repeatedly.	If team orders ketamine at 1–2 mg/kg for an awake look, vitals remain unchanged. (A). If patients are sedated with anything other than ketamine, they will desaturate by 5% every fifteen seconds during each intubation attempt, down to 60% (B). Vitals will return to (A) if patients are properly bag-valve-mask ventilated during attempts. Airway is unable to be secured without bronchoscopy or a cricothyrotomy. Team members are told their tube has been malpositioned if they manage to intubate the task trainer or manikin through direct or video laryngoscopy. (C). Once patient undergoes successful bronchoscopy or cricothyrotomy, oxygen saturation improves. If patient is not immediately ventilated (within ten seconds) of hitting a 60% oxygen saturation, patient will go into Vfib and will stay there until airway is secured (D). Team is brought back to the full-body manikin for advanced cardiopulmonary resuscitation. Team member may continue nasopharyngeal or oropharyngeal airway attempts on the airway task trainer while other team members perform cricothyrotomy, chest compressions, and/or medication administration on the manikin Return of spontaneous circulation is achieved once an airway is secured (C). Case ends after airway is secured and the Intensive Care Unit (ICU) is contacted for admission (or minute 15/facilitator’s discretion). If the team does not ask to call for transfer to a burn center’s ICU, the nurse should prompt, “We need this bed; triage is full. Do we need to do anything else?” If there is extra time during the case, can go to vitals E after airway is secured. Team should ensure there is no tension pneumothorax and then consider other causes of hypotension (cyanide toxicity, hypovolemia).	B: T 99.8°F HR 140 BP 140/96 RR 24 O_2_ sat 60% (on nasal cannula if left on for apneic oxygenation or room air) C: T 99.8°F HR 100 BP 140/96 RR 24O_2_ sat 94% on bag-valve mask or ventilator D: T 99.8°F HR–- BP–- RR - O_2_ sat undetectable E: T 99.8°F HR 120 BP 90/60 RR 24 O_2_ sat 94% on bag-valve mask or ventilator

**Figure:**
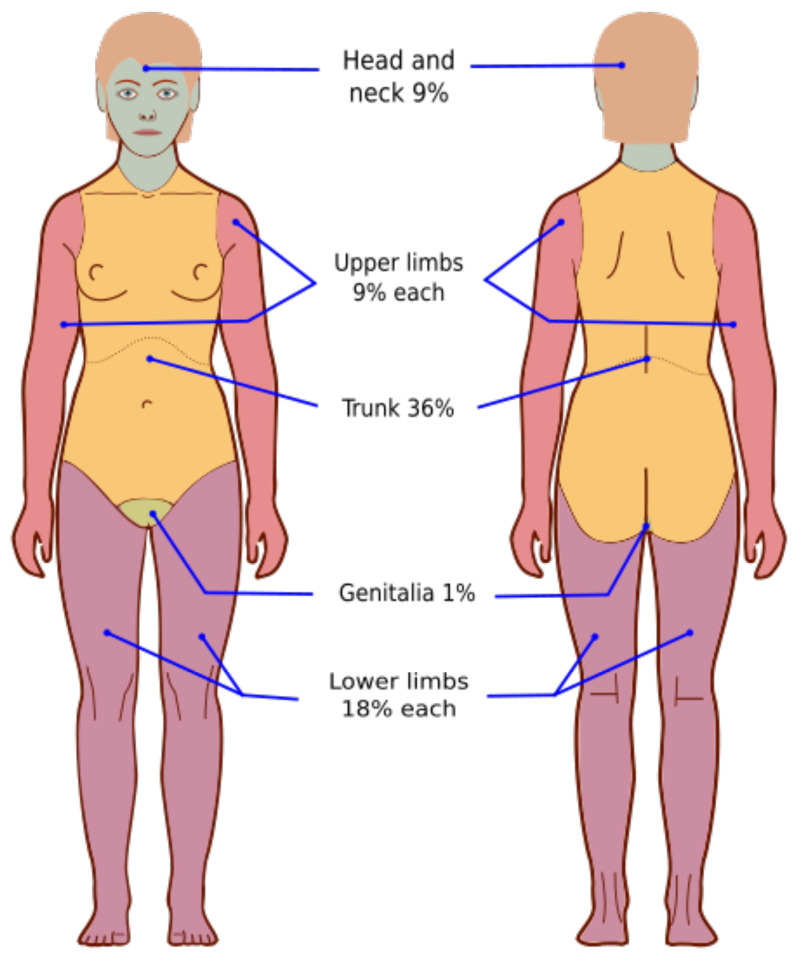


**Figure:**
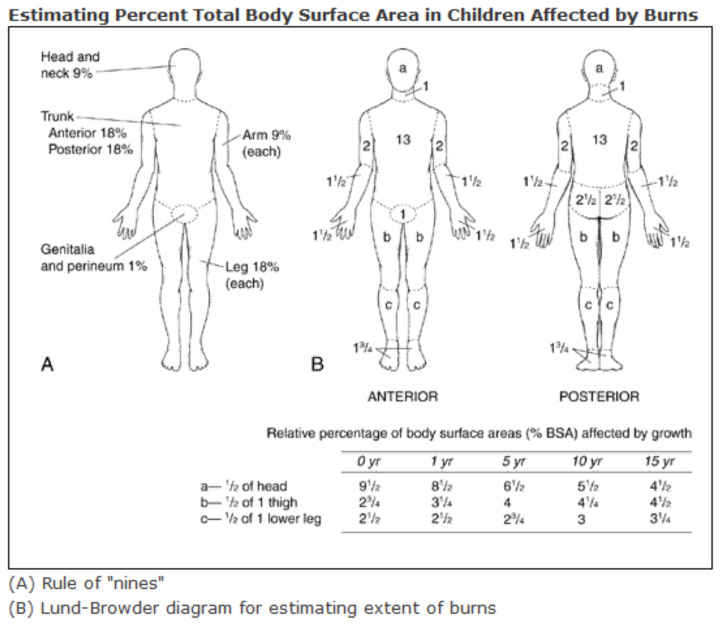


**Table t17-jetem-8-4-s49:** 

**Critical Actions:** Look into the patient’s mouth prior to intubating Call for help with the airway (surgery, otolaryngology, or anesthesia) Pre-oxygenate the patient with both nasal cannula set to 15 liters and a non-rebreather set to flush (all the way up until the knob will not turn any higher) Sedate the patient without using paralytics Verbalize a stepwise airway management plan prior to first attempt, including three next-step strategies Ensure airway equipment is at beside prior to first attempt, including suction, endotracheal tube with appropriate stylet, bag-valve mask, primary intubation equipment (laryngoscope blade with working light, video laryngoscopy), and equipment for at least three next-step airway strategies (video laryngoscopy, bougie, bronchoscope, laryngeal mask airway, scalpel). Admit to the intensive care unit at a regional burn center	0:00

**Table t18-jetem-8-4-s49:** 

	Milestone	Did not achieve level 1	Level 1	Level 2	Level 3
1	**Emergency Stabilization (PC1)**	□ Did not achieve Level 1	□ Recognizes abnormal vital signs	□ Recognizes an unstable patient, requiring intervention Performs primary assessment Discerns data to formulate a diagnostic impression/plan	□ Manages and prioritizes critical actions in a critically ill patient Reassesses after implementing a stabilizing intervention
2	**Performance of focused history and physical (PC2)**	□ Did not achieve Level 1	□ Performs a reliable, comprehensive history and physical exam	□ Performs and communicates a focused history and physical exam based on chief complaint and urgent issues	□ Prioritizes essential components of history and physical exam given dynamic circumstances
3	**Diagnostic studies (PC3)**	□ Did not achieve Level 1	□ Determines the necessity of diagnostic studies	□ Orders appropriate diagnostic studies. Performs appropriate bedside diagnostic studies/procedures	□ Prioritizes essential testing Interprets results of diagnostic studies Reviews risks, benefits, contraindications, and alternatives to a diagnostic study or procedure
4	**Diagnosis (PC4)**	□ Did not achieve Level 1	□ Considers a list of potential diagnoses	□ Considers an appropriate list of potential diagnosis May or may not make correct diagnosis	□ Makes the appropriate diagnosis Considers other potential diagnoses, avoiding premature closure
5	**Pharmacotherapy (PC5)**	□ Did not achieve Level 1	□ Asks patient for drug allergies	□ Selects an medication for therapeutic intervention, consider potential adverse effects	□ Selects the most appropriate medication and understands mechanism of action, effect, and potential side effects Considers and recognizes drug-drug interactions
6	**Observation and reassessment (PC6)**	□ Did not achieve Level 1	□ Reevaluates patient at least one time during case	□ Reevaluates patient after most therapeutic interventions	□ Consistently evaluates the effectiveness of therapies at appropriate intervals
7	**Disposition (PC7)**	□ Did not achieve Level 1	□ Appropriately selects whether to admit or discharge the patient	□ Appropriately selects whether to admit or discharge Involves the expertise of some of the appropriate specialists	□ Educates the patient appropriately about their disposition Assigns patient to an appropriate level of care (ICU/Tele/Floor) Involves expertise of all appropriate specialists
9	**General Approach to Procedures (PC9)**	□ Did not achieve Level 1	□ Identifies pertinent anatomy and physiology for a procedure Uses appropriate Universal Precautions	□ Obtains informed consent Knows indications, contraindications, anatomic landmarks, equipment, anesthetic and procedural technique, and potential complications for common ED procedures	□ Determines a back-up strategy if initial attempts are unsuccessful Correctly interprets results of diagnostic procedure
20	**Professional Values (PROF1)**	□ Did not achieve Level 1	□ Demonstrates caring, honest behavior	□ Exhibits compassion, respect, sensitivity and responsiveness	□ Develops alternative care plans when patients’ personal beliefs and decisions preclude standard care
22	**Patient centered communication (ICS1)**	□ Did not achieve level 1	□ Establishes rapport and demonstrates empathy to patient (and family) Listens effectively	□ Elicits patient’s reason for seeking health care	□ Manages patient expectations in a manner that minimizes potential for stress, conflict, and misunderstanding. Effectively communicates with vulnerable populations, (at risk patients and families)
23	**Team management (ICS2)**	□ Did not achieve level 1	□ Recognizes other members of the patient care team during case (nurse, techs)	□ Communicates pertinent information to other healthcare colleagues	□ Communicates a clear, succinct, and appropriate handoff with specialists and other colleagues Communicates effectively with ancillary staff

## INSTRUCTOR MATERIALS

**Case Title:** Inhalational Injury Secondary to House Fire

**Case Description & Diagnosis (short synopsis):** Patient is a 42-year-old male who presents by EMS to your community hospital (not a burn center) after a house fire. Per EMS, neighbors called 911 when they witnessed smoke. He was found unconscious lying on a couch without signs of blunt trauma. He awoke shortly after extrication from the building and was placed on a non-rebreather mask for shortness of breath. The patient is a full-body manikin lying on a cot, dressed in tear-away clothes that have been previously burned. Burn moulage is scattered over the entire body, including the face.

Participants should initiate a primary survey of the patient, which demonstrates that the patient is speaking in one- to two-word sentences with stridor. At this point, concern for impending airway collapse should be voiced, and the team should proceed with securing the airway, ideally with an initial awake look with ketamine. If surgery/otolaryngology/anesthesia are called, they are in the operating room and are not immediately available.

Once the decision was made to proceed with airway evaluation and management, the respiratory therapist moved the learners to a side table with the burned airway task trainer with a non-rebreather mask in place, which had been previously covered with a sheet. The table was in a location where the team members could still visualize the patient’s vital signs. The team was able to perform direct laryngoscopy, video laryngoscopy, and bronchoscopy on the burned airway task trainer, which has anatomical landmarks that are difficult to visualize due to edema and soot. Attempts at intubation were unsuccessful unless the patient underwent bronchoscopy, with precipitous falls in oxygen saturation during any prolonged attempt. If the residents moved towards cricothyrotomy, they were directed back to the whole-body manikin which had this capability.

Once the airway is secured, surgery/otolaryngology/anesthesia will return pages and be able to see the patient in the emergency department. If there is time left over in the case, the patient will become hypotensive to prompt the residents to consider cyanide toxicity. The patient should ultimately be transferred to the intensive care unit at a hospital with a dedicated burn center.

**Equipment or Props Needed:**

- High fidelity simulation mannequin (authors used a SimMan^®^ 3G Manikin)
- Burned airway task trainer (eg: 7-SIGMA Modular Burn Victim Skills Partial Airway Trainer) on a side table initially under a bedsheet
- Angiocatheters for peripheral intravenous access: 18 gauge, 20 gauge, 22 gauge
- Cardiac monitor
- Pulse oximetry
- IV pole
- Bilevel positive airway pressure (BiPAP) or continuous positive airway pressure (CPAP) mask
- Non-rebreather mask x2 (one on the full-body manikin, one on the airway task trainer)
- Nasal cannula x2 (one for use on the full-body manikin, one for use on the airway task trainer)
- Bag-valve mask x2 (one for use on the full-body manikin, one for use on the airway task trainer)
- Wound moulage to mock up soot/burns
- Tear-away/Velcro-ed clothes with areas of burns (achieved by burning specific areas of clothing then holding the clothes over a bonfire to add a smoky scent, stored in airtight bins until used for simulation)
- Normal saline (1 liter x2)
- Lactated Ringers (1 liter x2)
- Simulated medications with labeling: tetanus vaccine, fentanyl, hydromorphone, ketamine, etomidate, succinylcholine, rocuronium, propofol

**Actors needed:**

*One actor as the primary nurse and one clinical respiratory therapist (or one confederate acting as the respiratory therapist). Faculty may call in overhead as the surgeon, otolaryngologist, or anesthesiologist.*

**Stimulus Inventory:** [Table t2-jetem-8-4-s49]

**Background and brief information:** Patient is a 42-year-old male who presents to your community ED (not a burn center) via EMS after a house fire.

**Initial presentation:** 42-year-old male lying supine on the cot, speaking in one- to two-word sentences at a time with stridor and a frequent weak cough

- Past medical history: none
- Past surgical history: none
- Medications: none
- Allergies: none
- Social history: smokes cigarettes
- Family history: noncontributory
- Vital signs:
  ○ HR – 120 beats per minute
  ○ Resp rate – 24 respirations per minute
  ○ Temp – 99.8° F
  ○ BP – 140/96 mmHg
  ○ Pulse ox – 90% on a 15-liter non-rebreather mask
- Weight: 100 kilograms

Assessment: Lying supine, dyspneic with stridor, only able to speak one or two words at a time, intermittently weakly coughing

**How the scene unfolds:** Patient is a 42-year-old male who presents by EMS to your community ED (not a burn center) after a house fire. Per EMS, neighbors called 9-1-1 when they witnessed smoke. He was found unconscious lying on a couch without signs of blunt trauma. He awoke shortly after extrication from the building and was placed on a non-rebreather mask for shortness of breath. The patient is a full-body manikin lying on a cot with a non-rebreather in place, dressed in tear-away/Velcro-ed clothes that have been previously burned. Burn moulage is scattered over the entire body, including the face. If asked, glucose was 100 mg/dL and initial oxygen saturation on room air was 85%.

Participants should initiate a primary survey of the patient, which demonstrates that the patient is speaking in one- to two-word sentences with stridor. At this point, concern for impending airway collapse should be voiced, and the team should proceed with securing the airway, ideally with an initial “awake look” with ketamine. If surgery/otolaryngology/anesthesia are called, they are in the operating room and are not immediately available.

Once the decision was made to proceed with airway evaluation and management, the respiratory therapist should enter the room and move the team to a side table with the burned airway task trainer with a non-rebreather mask in place, which had been previously covered with a sheet. The table is in a location where the team members can still readily visualize the patient’s vital signs in real time. One of the simulation facilitators is in the room with the team to closely observe airway management techniques. If the residents request bronchoscopy, the respiratory therapist should leave the room to obtain this to reflect that specialized equipment may not be readily available at bedside. The residents are able to perform direct laryngoscopy, video laryngoscopy, and bronchoscopy on the burned airway task trainer, which has anatomy which is difficult to visualize due to edema and soot. Attempts at intubation will be unsuccessful unless patient undergoes bronchoscopy, with precipitous falls in oxygen saturation during any prolonged attempt. If residents themselves do not notice or verbalize a falling oxygen saturation, the respiratory therapist should verbalizie this out loud, eg, “You’re at 80 percent… you’re at 75%.” If the residents move towards cricothyrotomy, they will be directed back to the whole-body manikin with this capability. If the patient falls to 60% oxygen saturation, the patient will go into ventricular fibrillation, and they will be directed back to the full-body manikin for advanced cardiovascular life support measures.

Once the airway is secured, surgery and/or anesthesia will return pages and be able to see the patient in the emergency department. If the patient was pulseless but the airway was then secured, return of spontaneous circulation will be achieved. If there is time left over in the case, the patient can become hypotensive to prompt the residents to consider cyanide toxicity. The patient should ultimately be transferred to the intensive care unit at a hospital with a dedicated burn center.

**Critical actions:**

1. Look into the patient’s mouth prior to intubating
2. Call for help with the airway (surgery, otolaryngology, or anesthesia)
3. Pre-oxygenate the patient with both nasal cannula set to 15 liters and a non-rebreather set to flush (all the way up until the knob will not turn any higher)
4. Sedate the patient without using paralytics
5. Verbalize a stepwise airway management plan prior to first attempt, including three next-step strategies
6. Ensure airway equipment is at beside prior to first attempt, including suction, endotracheal tube with appropriate stylet, bag-valve mask, primary intubation equipment (laryngoscope blade with working light, video laryngoscopy), and equipment for at least three next-step airway strategies (video laryngoscopy, bougie, bronchoscope, laryngeal mask airway, scalpel).
7. Admit to the intensive care unit at a regional burn center

**Case Title:** Inhalational Injury Secondary to House Fire

**Chief Complaint:** Shortness of breath[Table t3-jetem-8-4-s49]

**General Appearance:** Lying supine, dyspneic with stridor, only able to speak one or two words at a time, intermittently weakly coughing

**Primary Survey:**

- **Airway:** stridor, dyspneic, only able to speak one or two words at a time, intermittently weakly coughing
- **Breathing:** diminished but symmetric bilaterally
- **Circulation:** heart regular tachycardic rhythm, 2+ symmetric pulses, capillary refill >3 seconds

**History:**

- **History of present illness:** Patient is a 42-year-old male who presents by EMS after a house fire. Per EMS, neighbors called 911 when they witnessed smoke. He was found unconscious lying on a couch without signs of blunt trauma. He awoke shortly after extrication from the building and was placed on a non-rebreather mask for shortness of breath.
  If asked:
  ○ Last tetanus immunization is unknown
  ○ There was no one else in the house
  ○ Glucose was 100 mg/dL
  ○ Initial oxygen saturation was 85% on room air
  ○ He was given 100 micrograms of fentanyl intravenously
- **Past medical history:** none
- **Past surgical history:** none
- **Medications:** none
- **Allergies:** none
- **Social history:** smokes cigarettes.
- **Family history:** noncontributory

**Vital Signs:**

- HR – 120
- Resp rate – 24
- Temp – 99.8° F
- BP – 140/96
- Pulse ox – 90% on a 15-liter non-rebreather mask
- Weight: 100 kg

**Assessment:** Lying supine, dyspneic with stridor, only able to speak one or two words at a time, intermittently weakly coughing

**Secondary Survey/Physical Examination:**

- **General appearance:** Lying supine, dyspneic with stridor, only able to speak one or two words at a time, intermittently weakly coughing
- **HEENT:**
  ○ **Head:** normocephalic, covered with scattered superficial and partial-thickness burns and soot
  ○ **Eyes:** scleral injection bilaterally, otherwise within normal limits
  ○ **Ears:** scattered burns, otherwise within normal limits
  ○ **Nose:** scattered burns, singed hairs, soot present in bilateral nares
  ○ **Throat / oropharynx:** soot present throughout, mildly edematous tongue, uvular edema present, tolerating secretions
- **Neck**: scattered burns, otherwise within normal limits
- **Heart**: regular tachycardic rhythm. Otherwise within normal limits
- **Lungs**: diminished breath sounds bilaterally symmetric, dyspneic, +accessory muscle use present
- **Abdominal / GI**: scattered burns, otherwise within normal limits
- **GU**: scattered burns, otherwise within normal limits
- **Rectal**: deferred/within normal limits
- **Extremities**: scattered burns, otherwise within normal limits with soft compartments throughout. No circumferential burns
- **Neuro**: within normal limits
- **Skin**: scattered superficial and partial thickness burns, otherwise within normal limits
- **Lymph**: within normal limits

*Electrocardiogram, sinus tachycardia*

Heilman, J. Sinus tachycardia as seen on ECG. In: Wikimedia Commons.

https://commons.wikimedia.org/wiki/File:Sinustachy.JPG. Published June 15, 2012. CC BY-SA 3.0.[Fig f1-jetem-8-4-s49]

*Chest X-ray*

Stillwaterising. Chest Xray PA 3-8-2010. In: Wikimedia Commons. File:Chest Xray PA 3-8-2010.png – Wikimedia Commons. Public domain.[Fig f2-jetem-8-4-s49]

*Chest X-ray, post endotracheal intubation*

Jain A, Patel A, Hoppe IC. Benzocaine-Induced Cyanosis. In: Open-i.

https://openi.nlm.nih.gov/detailedresult?img=PMC4879862_eplasty16ic18_fig2&query=&req=4 Public domain.[Fig f3-jetem-8-4-s49][Table t4-jetem-8-4-s49][Table t5-jetem-8-4-s49][Table t6-jetem-8-4-s49][Table t7-jetem-8-4-s49][Table t8-jetem-8-4-s49][Table t9-jetem-8-4-s49][Table t10-jetem-8-4-s49][Table t11-jetem-8-4-s49][Table t12-jetem-8-4-s49][Table t13-jetem-8-4-s49][Table t14-jetem-8-4-s49][Table t15-jetem-8-4-s49]

## OPERATOR MATERIALS

[Table t16-jetem-8-4-s49]

**Diagnosis:**

Inhalational Injury Secondary to House Fire

**Disposition:**

ICU at a regional burn center

## DEBRIEFING AND EVALUATION PEARLS

### Inhalational Injury from Burns: Learning Pearls

- Indications for early intubation:^3^
  ○ Signs of airway obstruction: hoarseness, stridor, accessory respiratory muscle use, sternal retraction
  ○ Extent of the burn (Total Body Surface Area [TBSA] burn > 40–50%)
  ○ Extensive and deep facial burns
  ○ Burns inside the mouth
  ○ Significant edema or risk for edema
  ○ Difficulty swallowing
  ○ Signs of respiratory compromise: inability to clear secretions, respiratory fatigue, poor oxygenation, or poor ventilation
  ○ Decreased level of consciousness where airway protective reflexes are impaired
  ○ Anticipated patient transfer of large burn with airway issue without qualified personnel to intubate en-route
- Inhalation injury patients are often intubated unnecessarily when they can instead be observed on supplemental oxygen. Some of the commonly taught indications for intubation include: Superficial partial-thickness facial burns, singed facial and nasal hairs, and flash burns from home oxygen. These alone are not indications for intubation.^3^ The decision to intubate should be made based on the provider’s discretion and the previously outlined indications, particularly shortness of breath and patients with large TBSA numbers.^4^ Pay close attention and re-assess the airway often.
- When intubating the inhalation injury patient, always anticipate and plan for a difficult airway. These patients will often have a significant amount of soot obscuring landmarks, which makes it difficult to visualize the vocal cords. They are also at risk of having extensive edema, which will not only distort landmarks but also make it difficult to pass an endotracheal (ET) tube. While an ET tube large enough to facilitate bronchoscopy would be ideal, the most important goal is to secure the airway. Have smaller ET tubes readily on hand. Edema will often continue to worsen in the initial phase of the inhalation injury, and this is one of the many reasons that timely intubation is so important.
- As with any airway, pre-oxygenation is an important step prior to intubation when time allows. Pre-oxygenation denitrogenates the lungs and builds up a reservoir of oxygen which extends safe apnea time. To achieve effective pre-oxygenation, administer oxygen via nasal cannula at 15 liters/minute while simultaneously administering flush rate oxygen via non-rebreather. In an ideal situation, this should be maintained for around 3 minutes prior to intubation.^5^
- Be aware of the risk of paralyzing a difficult airway. You are completely removing that patient’s respiratory drive for an extended period of time. If you are unable to secure the airway or bag the patient adequately, they will die. In these cases, consider performing an awake intubation. This process uses topical anesthetics and sedatives without neuromuscular blockade in order to preserve the patient’s respiratory drive while you attempt intubation.
- Ketamine is a good choice during an anticipated difficult airway and can be used to perform an awake intubation. When administered in appropriate dosages (0.25 to 0.5 mg/kg intravenously every ten minutes, titrated up as needed) it will provide adequate sedation while maintaining the patient’s respiratory drive.^5^
- Prior to intubation, it’s important to announce to your team what your plan is from start to finish. You should explain what your initial approach will be. You should then verbalize your back-up options, up to and including a surgical airway. This will mentally prepare your team for this possibility, and if it does reach that point, they will be better equipped to handle it.
- Burn injuries that should be referred to a burn center:^3^
  ○ Partial thickness burns greater than 10% TBSA.
  ○ Burns that involve the face, hands, feet, genitalia, perineum, or major joints.
  ○ Third-degree (full-thickness) burns in any age group.
  ○ Electrical burns, including lightening injury.
  ○ Chemical burns.
  ○ Inhalation injury.
  ○ Burn injury in patients with preexisting medical disorders that could complicate management, prolong recovery, or affect mortality.
  ○ Any patients with burns and concomitant trauma (such as fractures) in which the burn injury poses the greatest risk of morbidity or mortality. In such cases, if the trauma poses the greater immediate risk, the patient may be stabilized initially in a trauma center before being transferred to a burn center. Physician judgment will be necessary in such situations and should be in concert with the regional medical control plan and triage protocols.
  ○ Burned children in hospitals without qualified personnel or equipment for the care of children.
  ○ Burn injury in patients who will require special social, emotional, or rehabilitative intervention.
- Other considerations in burn patients:
  ○ All severe burn patients should be evaluated per Advanced Trauma Life Support guidelines.
  ○ Carbon monoxide toxicity^5^
    ■ Carbon monoxide exposure in the setting of burn patients occurs due to the incomplete combustion of carbon containing products. Carbon monoxide binds to hemoglobin with a much stronger affinity than oxygen, forming carboxyhemoglobin. This ultimately prevents the transport of oxygen and results in tissue hypoxia.
    ■ Presentation is often nonspecific, with the most common symptom being headaches. In minor cases, patients may experience malaise, nausea, dizziness, myalgias. More severe cases can present with coma, seizures, hypotension, cardiac arrest, metabolic acidosis.
    ■ Pulse oximetry will read as normal, can confirm with co-oximetry.
    ■ Treatment involves high flow oxygen administered via non-rebreather. The primary indication for hyperbaric oxygen is to prevent long-term neurologic sequelae.
  ○ Cyanide toxicity^5^
    ■ In the context of fires, can be produced from the burning of plastics and upholstery. Inhibits oxidative metabolism by binding to cytochrome C-oxidase within the electron transport train. Adenosine triphosphate (ATP) is depleted and poisoned tissue ceases to function.
    ■ Can have a broad presentation, but severe cases present with coma, seizures, dysrhythmias, cardiovascular collapse.
    ■ Pulse ox readings will be accurate. pO2 on a venous blood gas will be elevated due to inability of tissue to utilize oxygen. Specific lab test for cyanide toxicity can take days to result, little clinical utility. Patients will have significantly elevated lactate.
    ■ If suspected, have a low threshold to administer treatment. First option is hydroxocobalamin, which rapidly binds cyanide and forms cyanocobalamin (B12). Second option is sodium thiosulfate, which helps convert cyanide to thiocyanate, which is excreted in urine.
- Rule of 9’s can be used to estimate TBSA of second degree and deeper burns, with the trunk and extremity values below representing summed totals for both anterior and posterior burns: for example, an anterior arm burn alone represents 4.5% TBSA, while a circumferential arm burn (anterior and posterior) represents 9% TBSA.^3^,^6^
- Lund Browder is used more commonly by burn centers to provide a more accurate assessment of TBSA^3^,^7^
- Multiple formulas exist to calculate fluid needs in the first 24 hours post-burn. These formulas are based on TBSA and weight. The two most popular formulas are the Parkland Formula (4 ml/kg/%TBSA/24 hours) and the Modified Brooke Formula (2 ml/kg/%TBSA/24 hours). The Parkland Formula has been shown to result in fluid over-resuscitation, and the American Burn Association currently recommends using the Modified Brooke Formula,^5^
- Fiberoptic intubation tips:
  ○ As you would with any video-assisted intubation, arrange your screen such that you do not have to turn your head/body while performing the scope. Since you will most likely be facing the patient, the screen should be at or behind the head of the bed.
  ○ Select the appropriate size scope. There are three sizes: slim (outer diameter [OD] 3.8mm, gray), regular (OD 5.0mm, green), and large (OD 5.8mm, orange). For our purposes, select the gray or the green so as to minimize trauma to the nasal turbinates or other laryngeal structures.
  ○ The scope should be oriented such that the lever is facing down and suction button facing up. Your dominant hand should hold the handle with your thumb on the lever and your pointer finger on the suction button. Attach the suction tubing to the connector located on the side of the handle. You will notice a working channel on the butt end of the handle. This is used mainly for bronchoalveolar lavage and rarely necessary for emergency medicine purposes. With your dominant hand on the handle, use your non-dominant hand to hold the distal end of the scope to keep the cord taut. Any slack in the scope can make it difficult to maneuver the camera.
  ○ When inserting the scope, advance perpendicular to the wall (or head of bed). This will pass the scope parallel to the turbinates. Be sure to not advance cephalad so as to minimize the risk of injuring the cribriform plate. For the purposes of fiberoptic nasotracheal intubation, you may pre-load a 6-0 endotracheal tube (ETT) on the scope OR blindly insert the ETT to roughly 14–15cm (termed “subtotal intubation”). In theory, this should place the tip of the ETT just above the glottis and help facilitate passage of the scope. Advance the scope until you visualize the carina. Recruit the help of an assistant to advance the ETT over the scope while holding the scope steady. Once the ETT is in place, inflate the balloon and remove the scope, slowly visualizing the tip of the ETT as you withdraw, thereby confirming placement.
- Closed loop communication and team interaction
  ○ Pointed roles during a critical event are extremely important in providing maximal care.
  ○ Address team members by their roles or names when asking for an action and request a verbal confirmation that you were heard and understood.

## SIMULATION ASSESSMENT

*Inhalational Injury Secondary to House Fire*

Learner: _________________________________________

***Assessment Timeline***

This timeline is to help observers assess their learners. It allows observer to make notes on when learners performed various tasks, which can help guide debriefing discussion.[Table t17-jetem-8-4-s49]

**Critical Actions:**

□ Look into the patient’s mouth prior to intubating
□ Call for help with the airway (surgery, otolaryngology, or anesthesia)
□ Pre-oxygenate the patient with both nasal cannula set to 15 liters and a non-rebreather set to flush (all the way up until the knob will not turn any higher)
□ Sedate the patient without using paralytics
□ Verbalize a stepwise airway management plan prior to first attempt, including three next-step strategies
□ Ensure airway equipment is at beside prior to first attempt, including suction, endotracheal tube with appropriate stylet, bag-valve mask, primary intubation equipment (laryngoscope blade with working light, video laryngoscopy), and equipment for at least three next-step airway strategies (video laryngoscopy, bougie, bronchoscope, laryngeal mask airway, scalpel).
□ Admit to the intensive care unit at a regional burn center

**Summative and formative comments:**

**Milestones assessment:** [Table t18-jetem-8-4-s49]
