# Supplementary material for: Inhalational Injury Secondary to House Fire
Source: J Educ Teach Emerg Med. 2023 Oct 31;8(4):S49–79. doi: 10.21980/J8TW7N (PMC10631807; doi:10.21980/J8TW7N)
Supplement: Supplementary file 1 [file jetem-8-4-s49-supp1.pptx]

## Slide 1
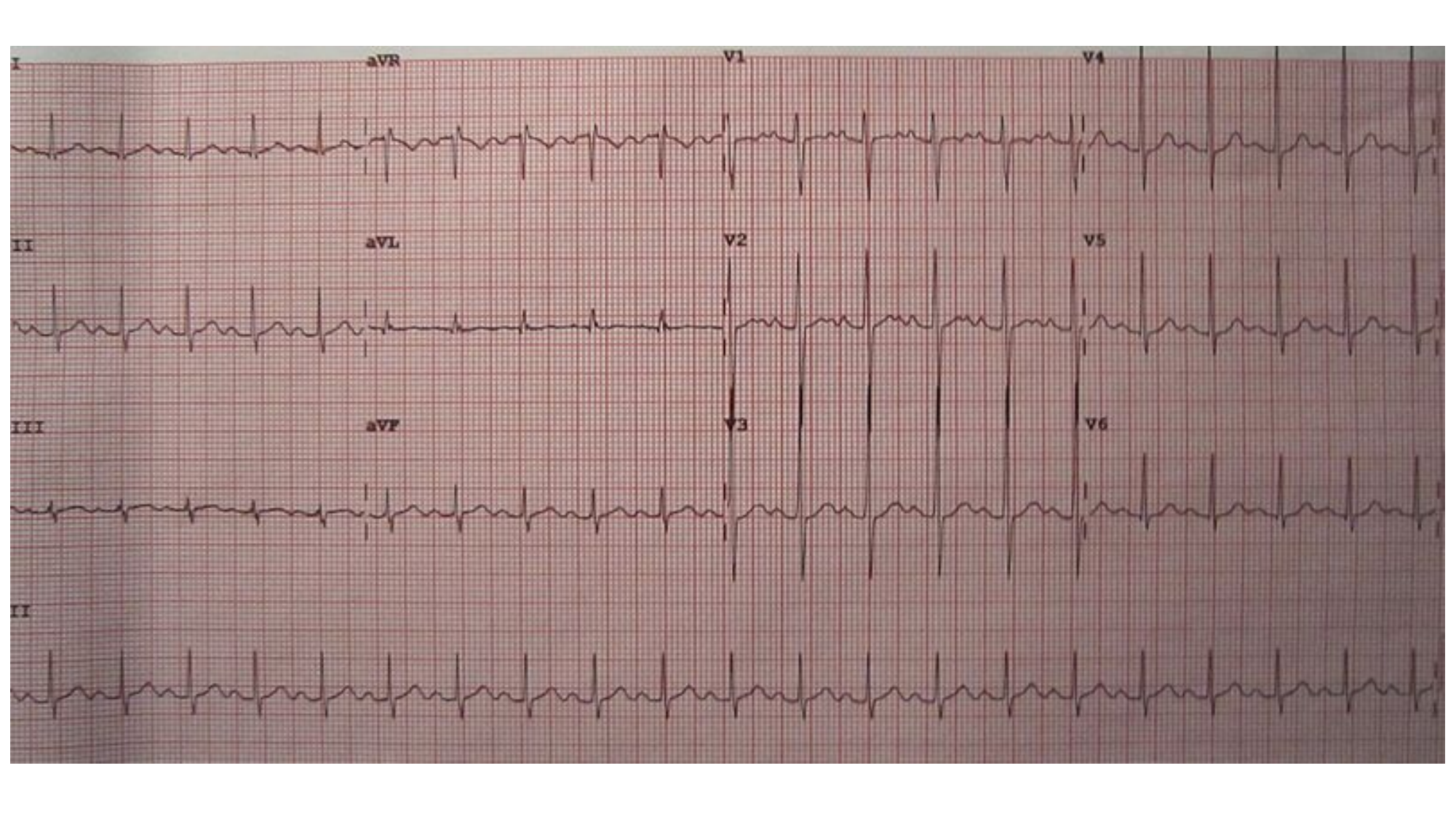

## Slide 2
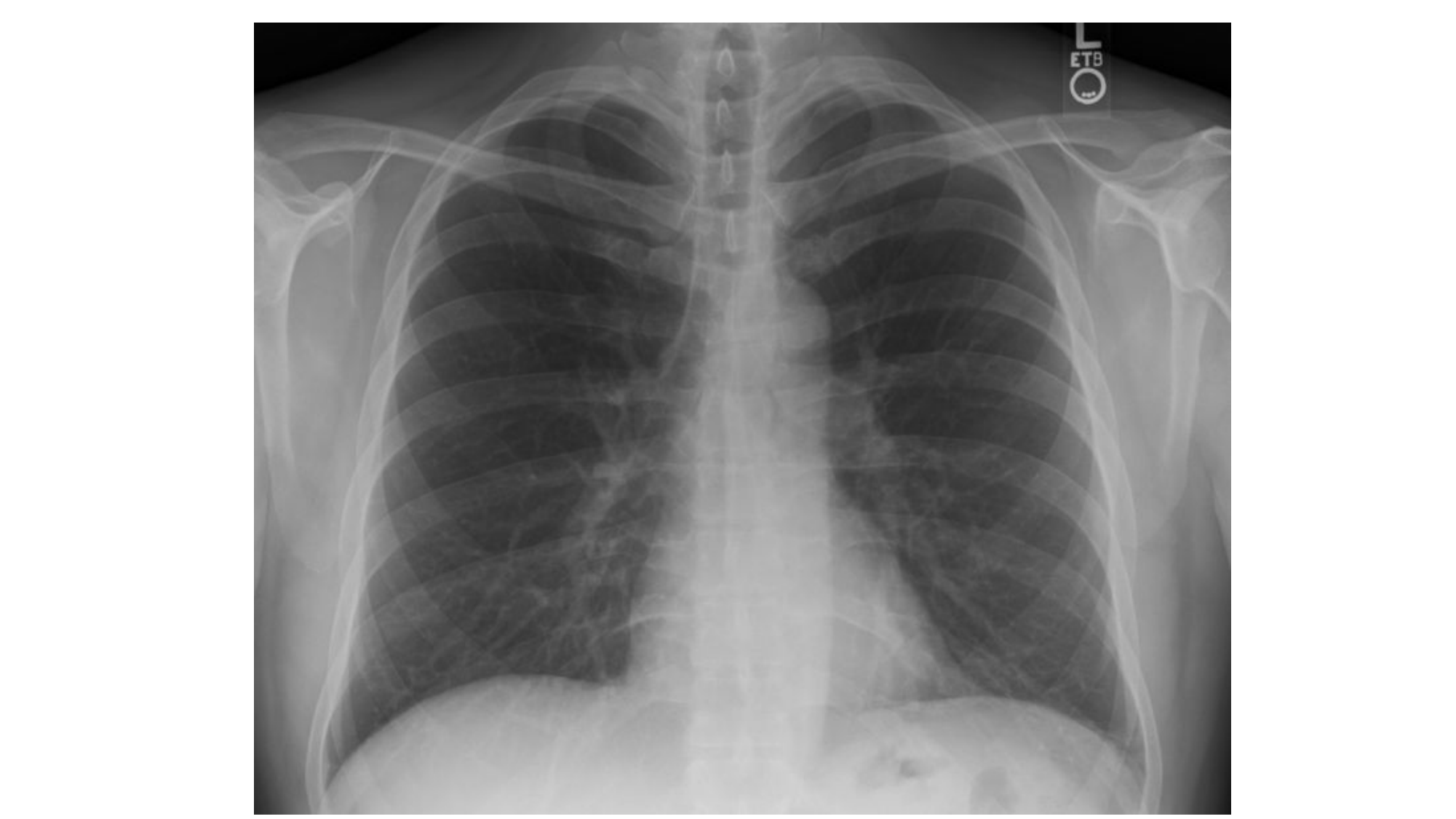

## Slide 3
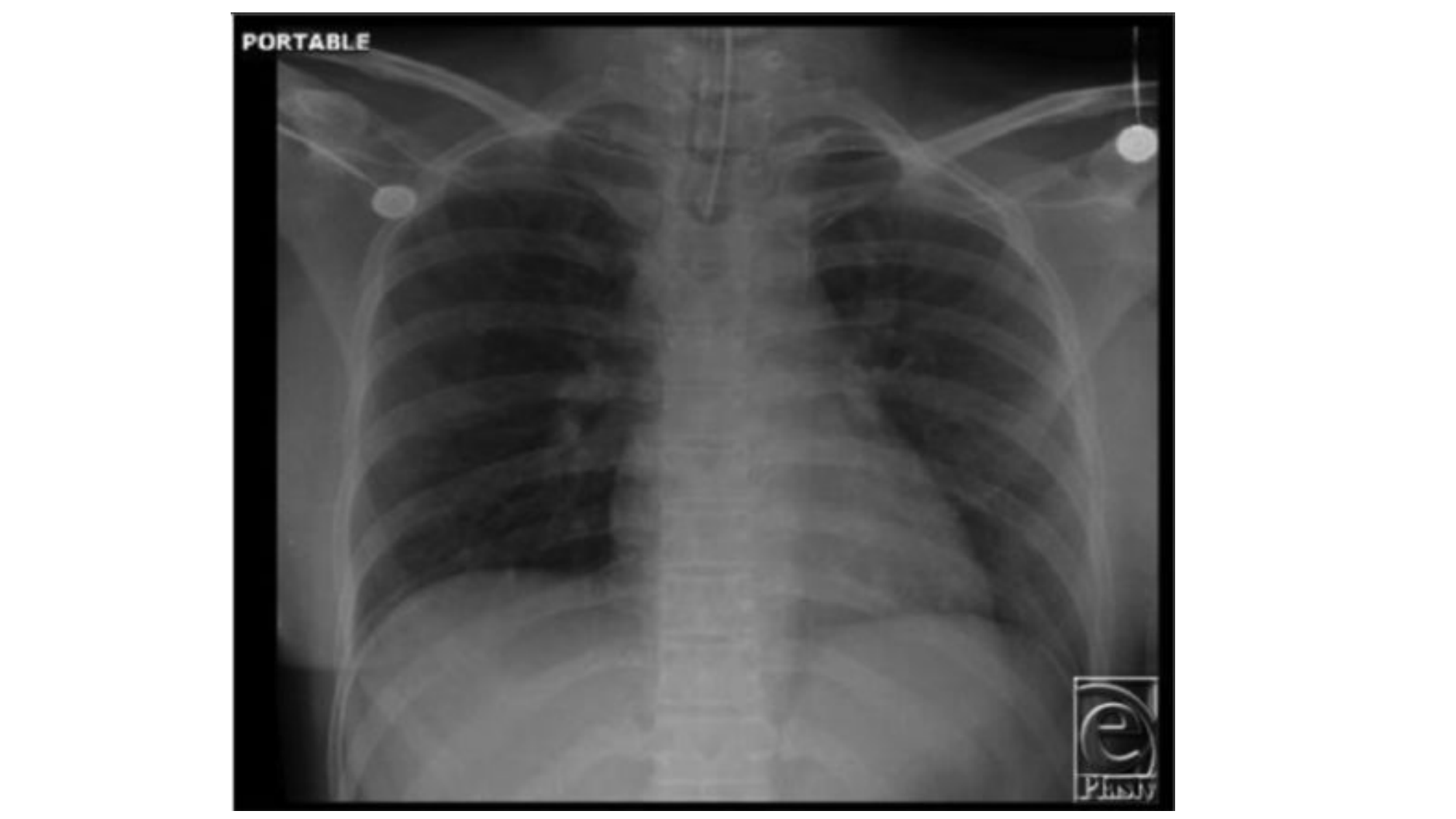

## Slide 4
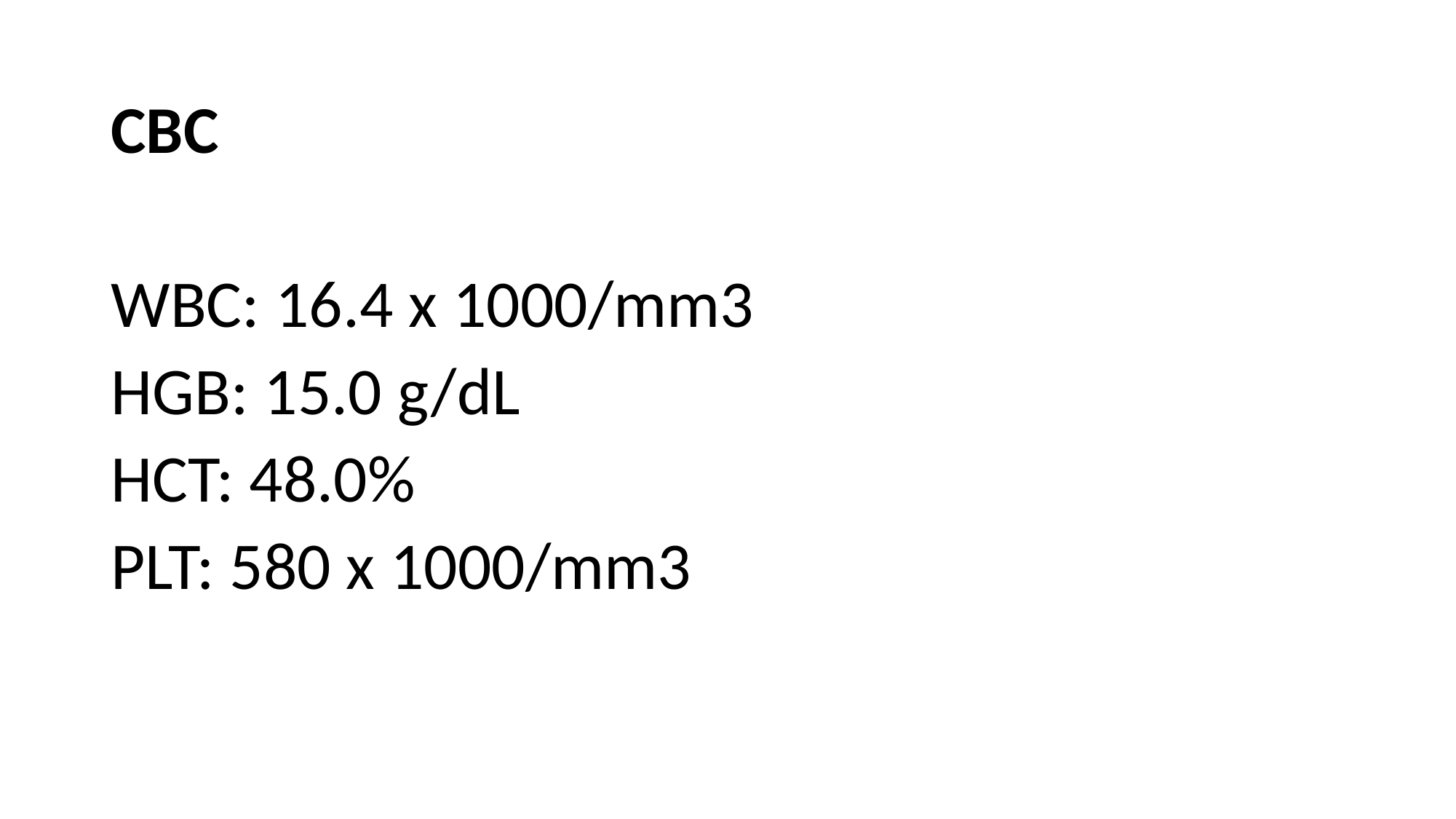

CBC
WBC: 16.4 x 1000/mm3
HGB: 15.0 g/dL
HCT: 48.0%
PLT: 580 x 1000/mm3

## Slide 5
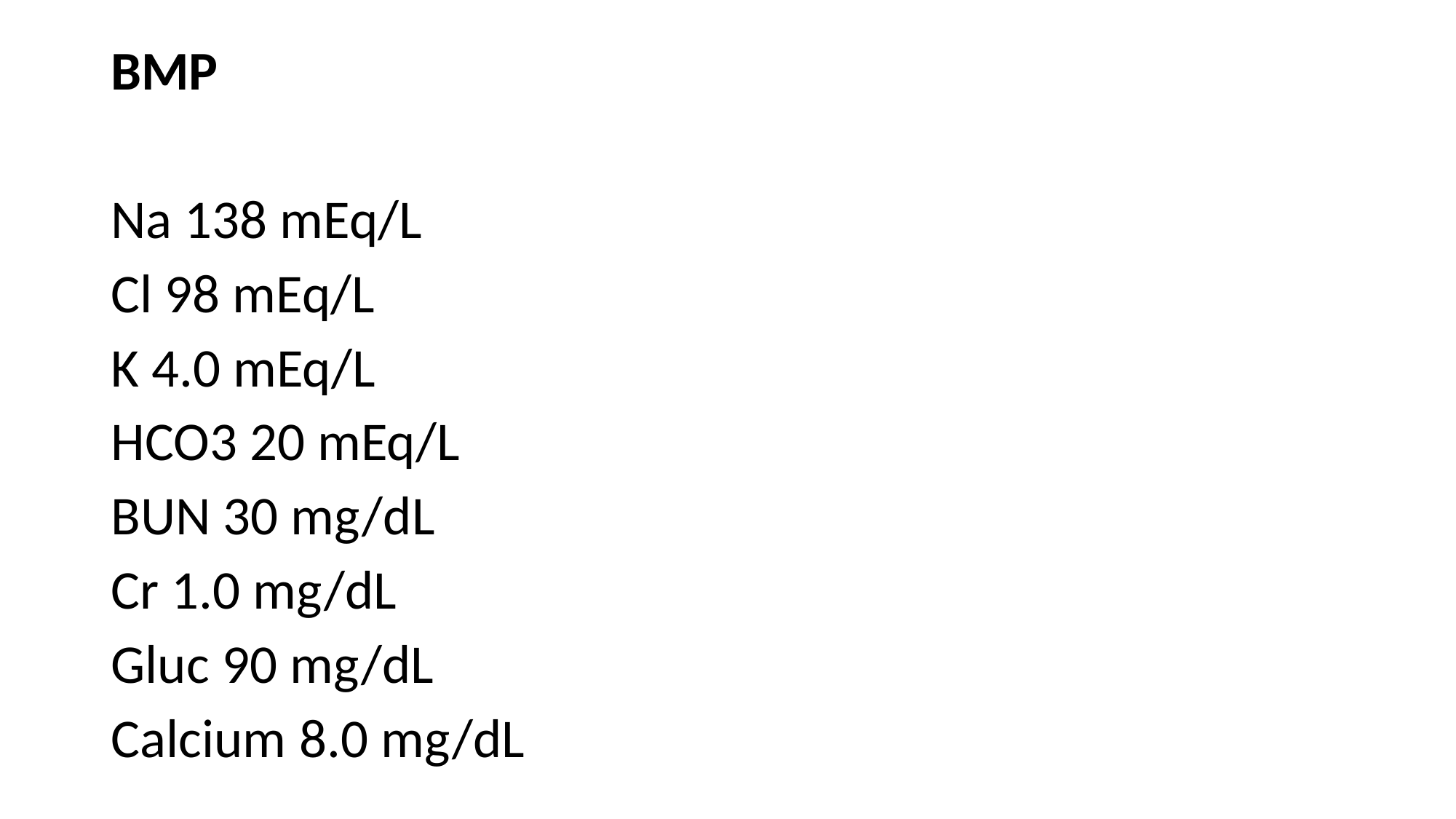

BMP
Na 138 mEq/L
Cl 98 mEq/L
K 4.0 mEq/L
HCO3 20 mEq/L
BUN 30 mg/dL
Cr 1.0 mg/dL
Gluc 90 mg/dL
Calcium 8.0 mg/dL

## Slide 6
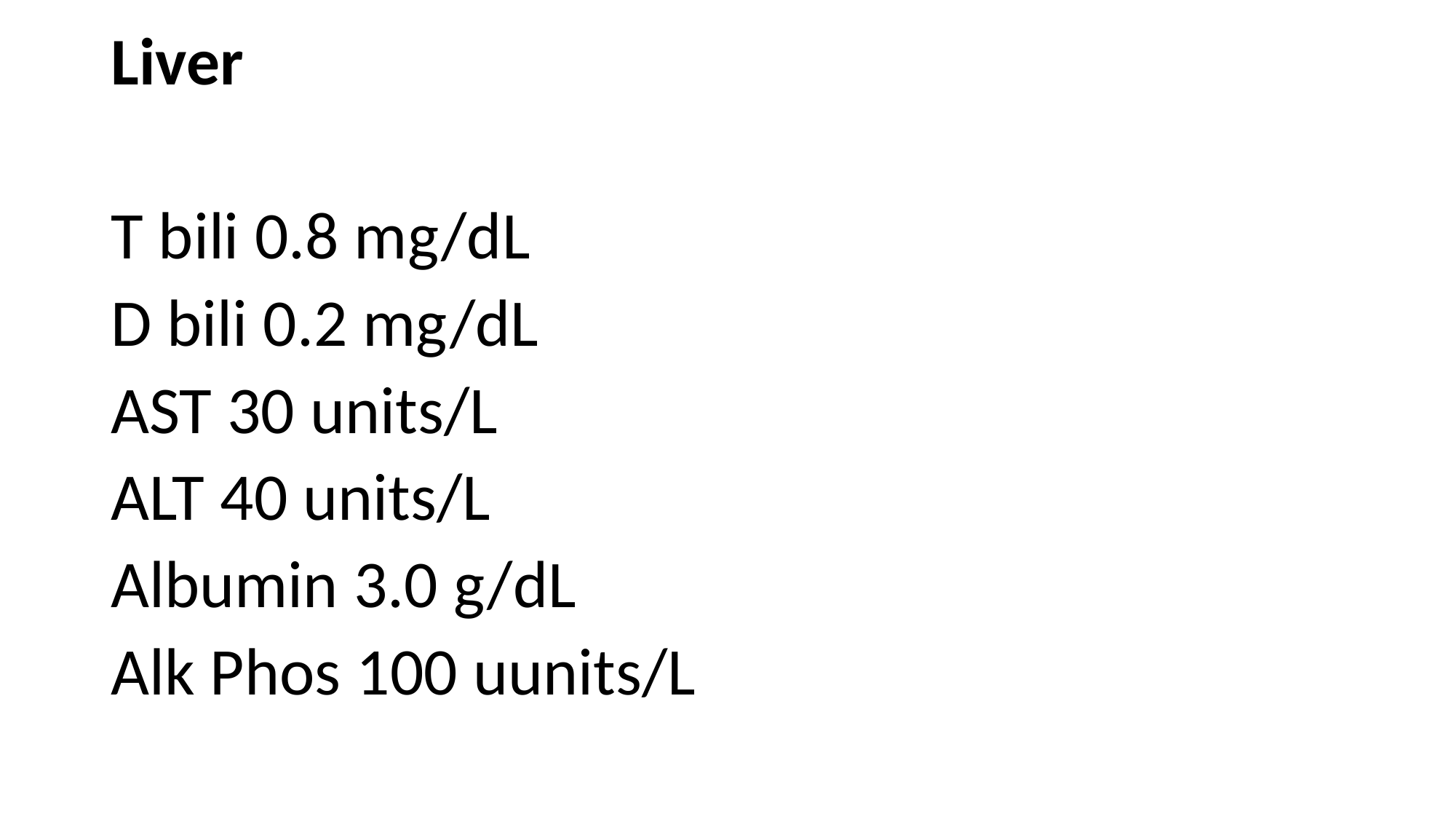

Liver
T bili 0.8 mg/dL
D bili 0.2 mg/dL
AST 30 units/L
ALT 40 units/L
Albumin 3.0 g/dL
Alk Phos 100 uunits/L

## Slide 7
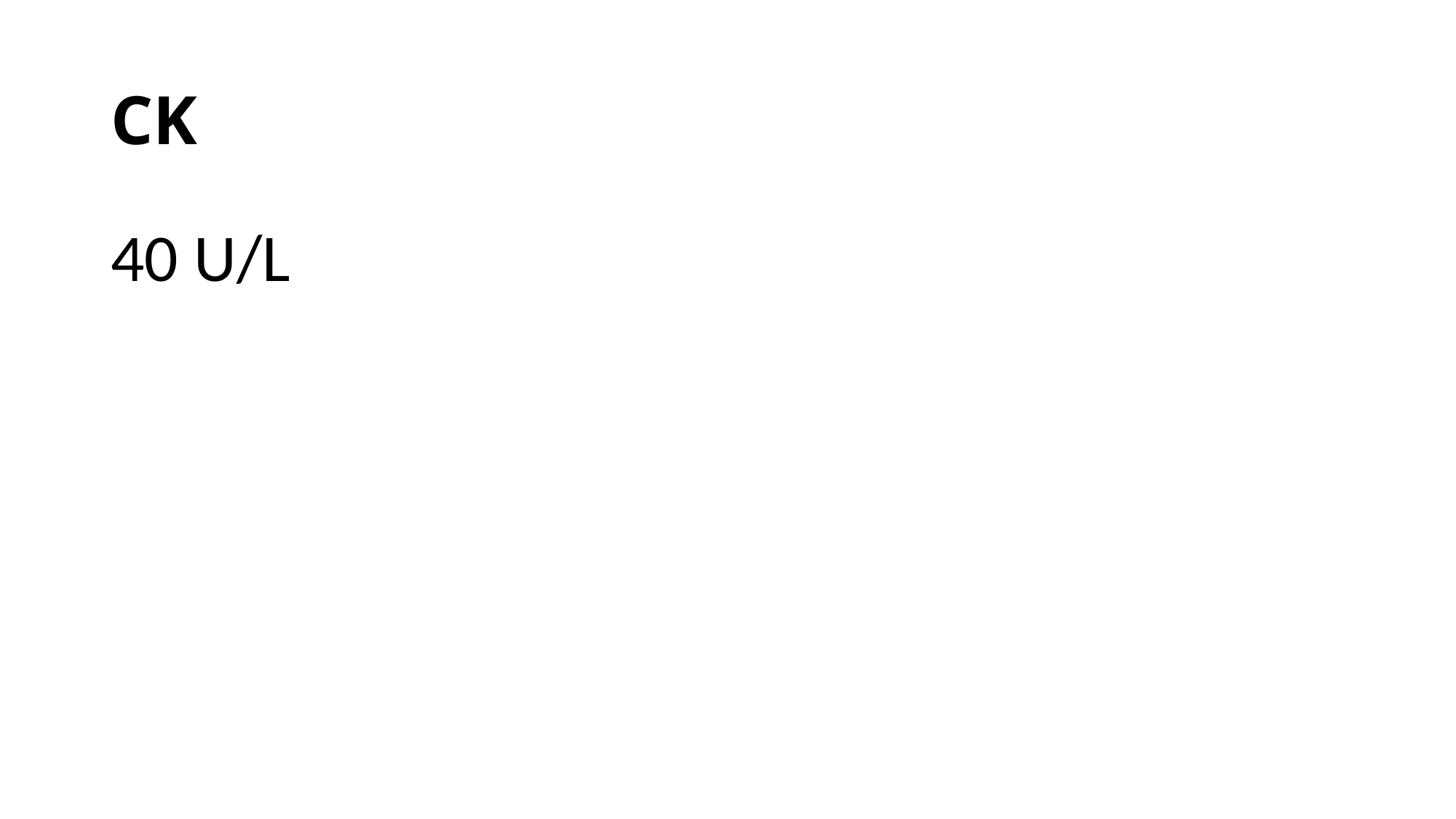

# CK
40 U/L

## Slide 8
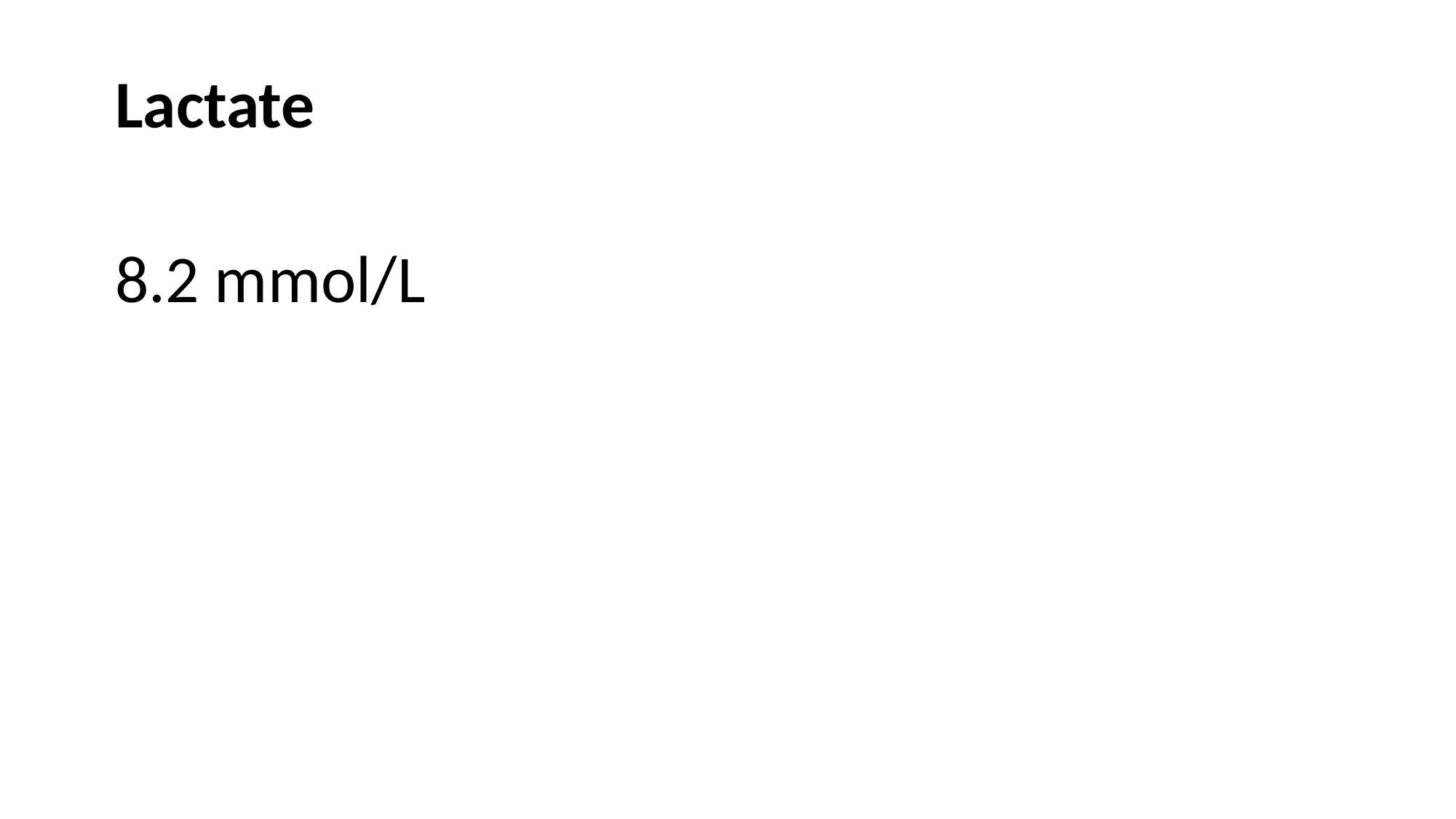

Lactate
8.2 mmol/L

## Slide 9
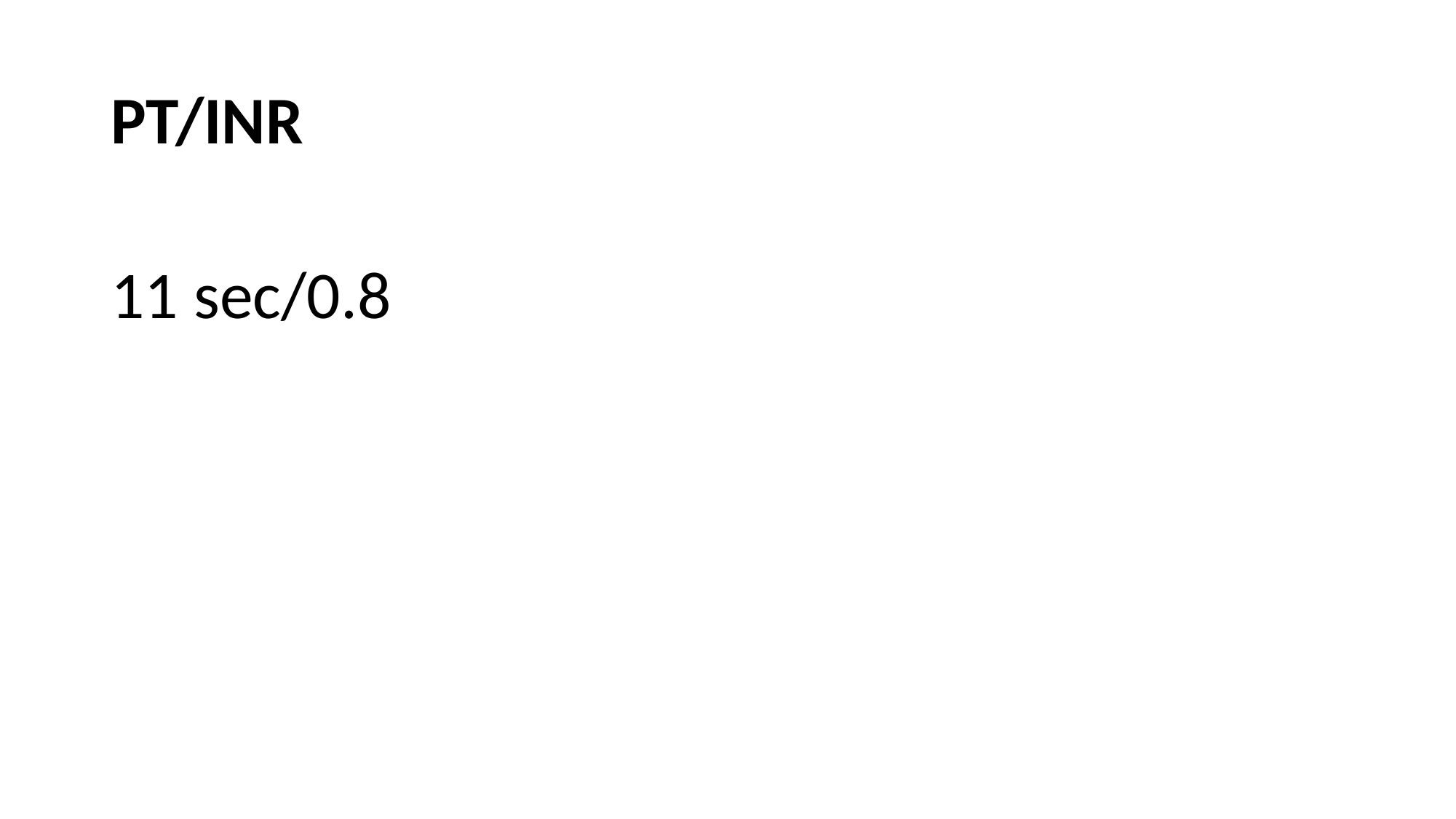

PT/INR
11 sec/0.8

## Slide 10
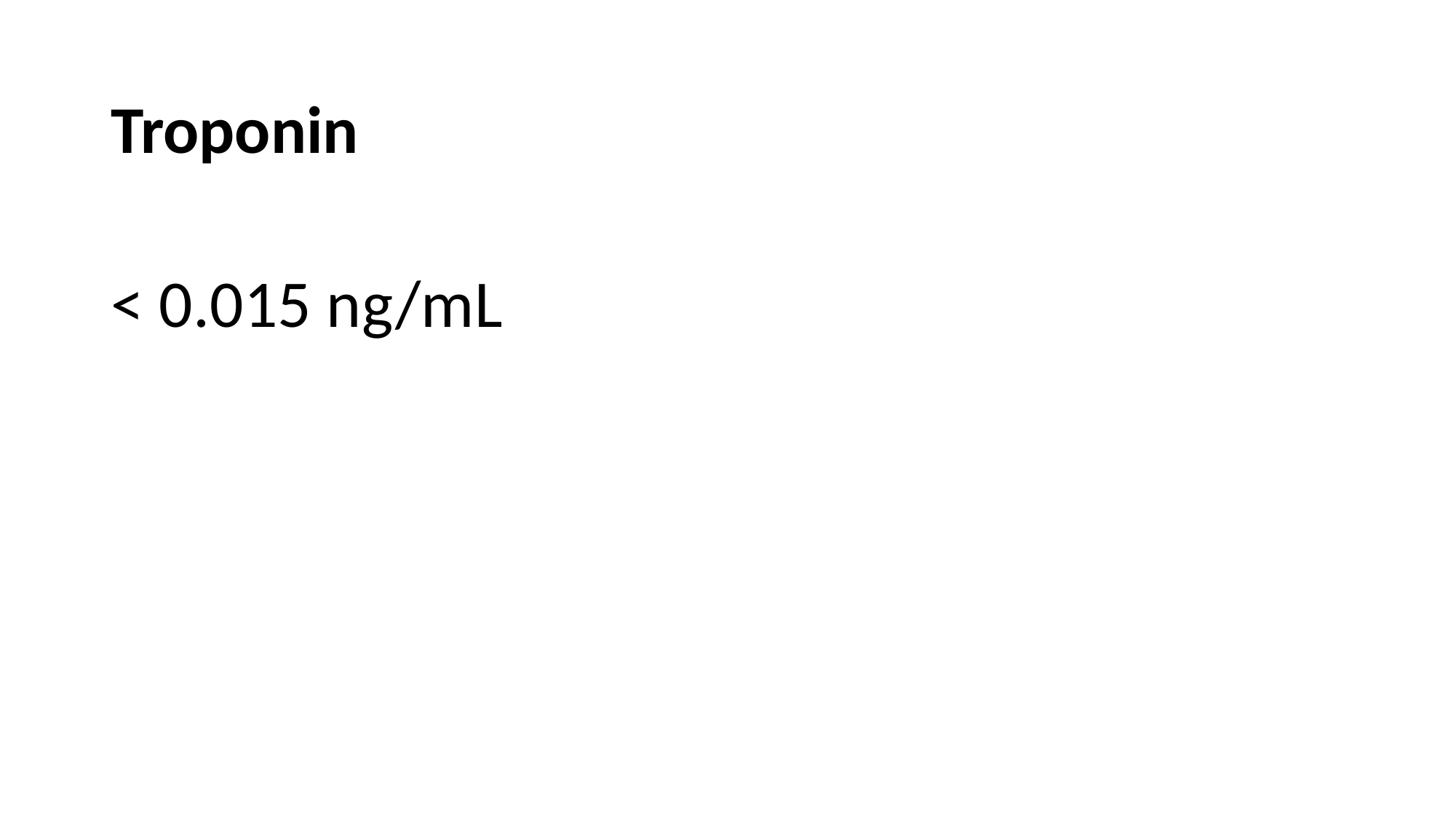

Troponin
< 0.015 ng/mL

## Slide 11
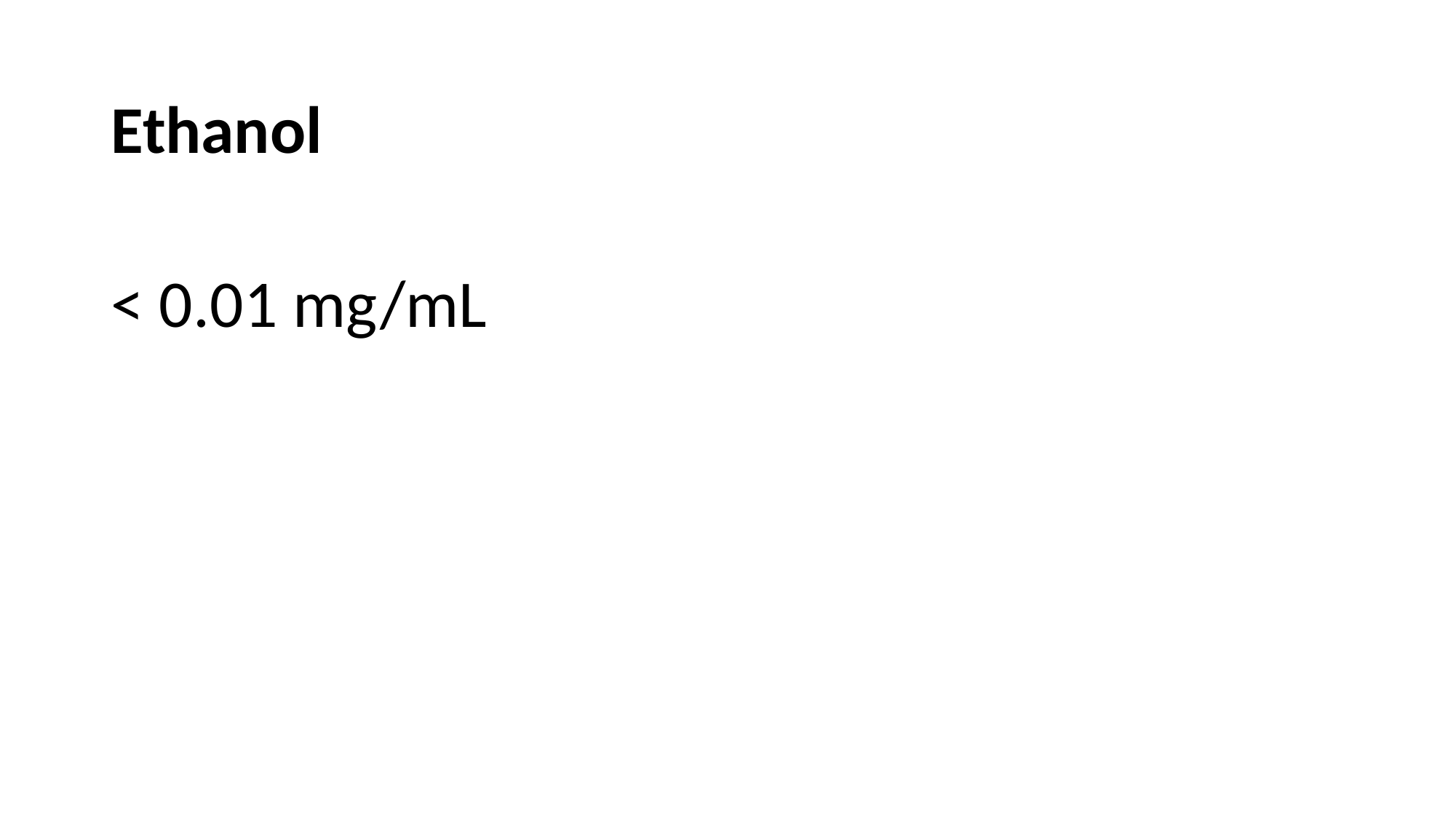

Ethanol
< 0.01 mg/mL

## Slide 12
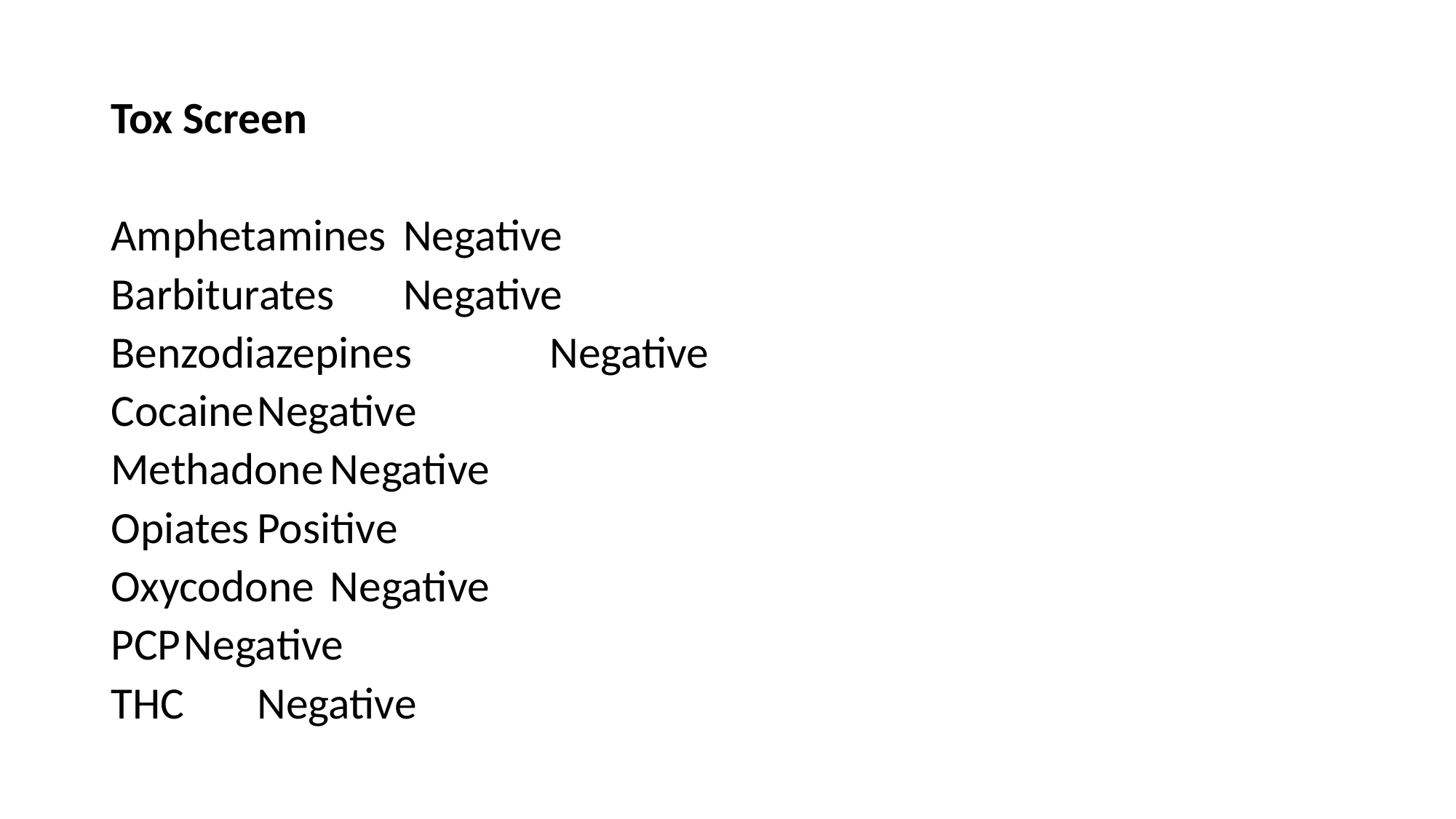

Tox Screen
Amphetamines			Negative
Barbiturates			Negative
Benzodiazepines		Negative
Cocaine				Negative
Methadone			Negative
Opiates				Positive
Oxycodone			Negative
PCP					Negative
THC					Negative

## Slide 13
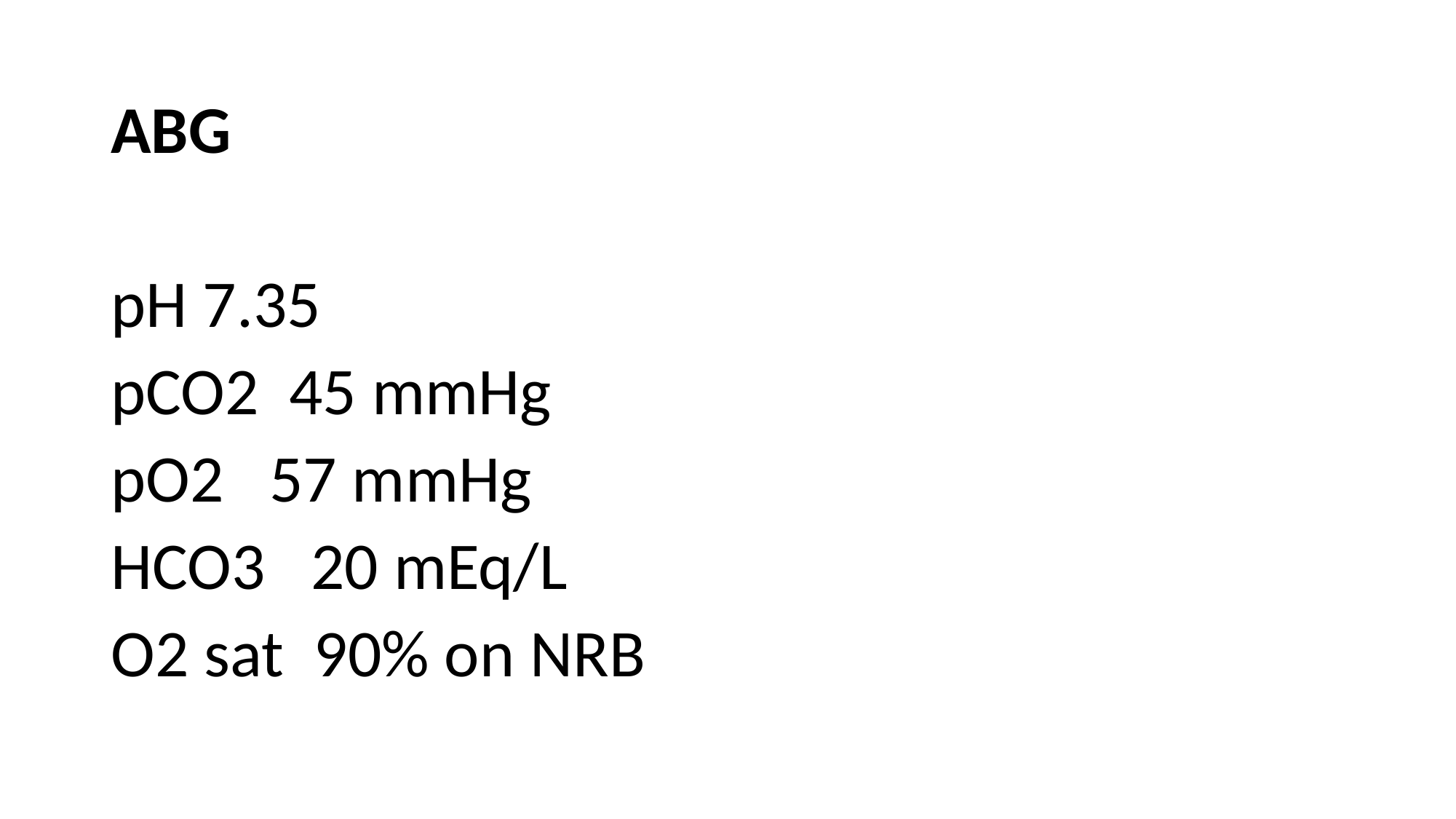

ABG
pH 7.35
pCO2 45 mmHg
pO2 57 mmHg
HCO3 20 mEq/L
O2 sat 90% on NRB

## Slide 14
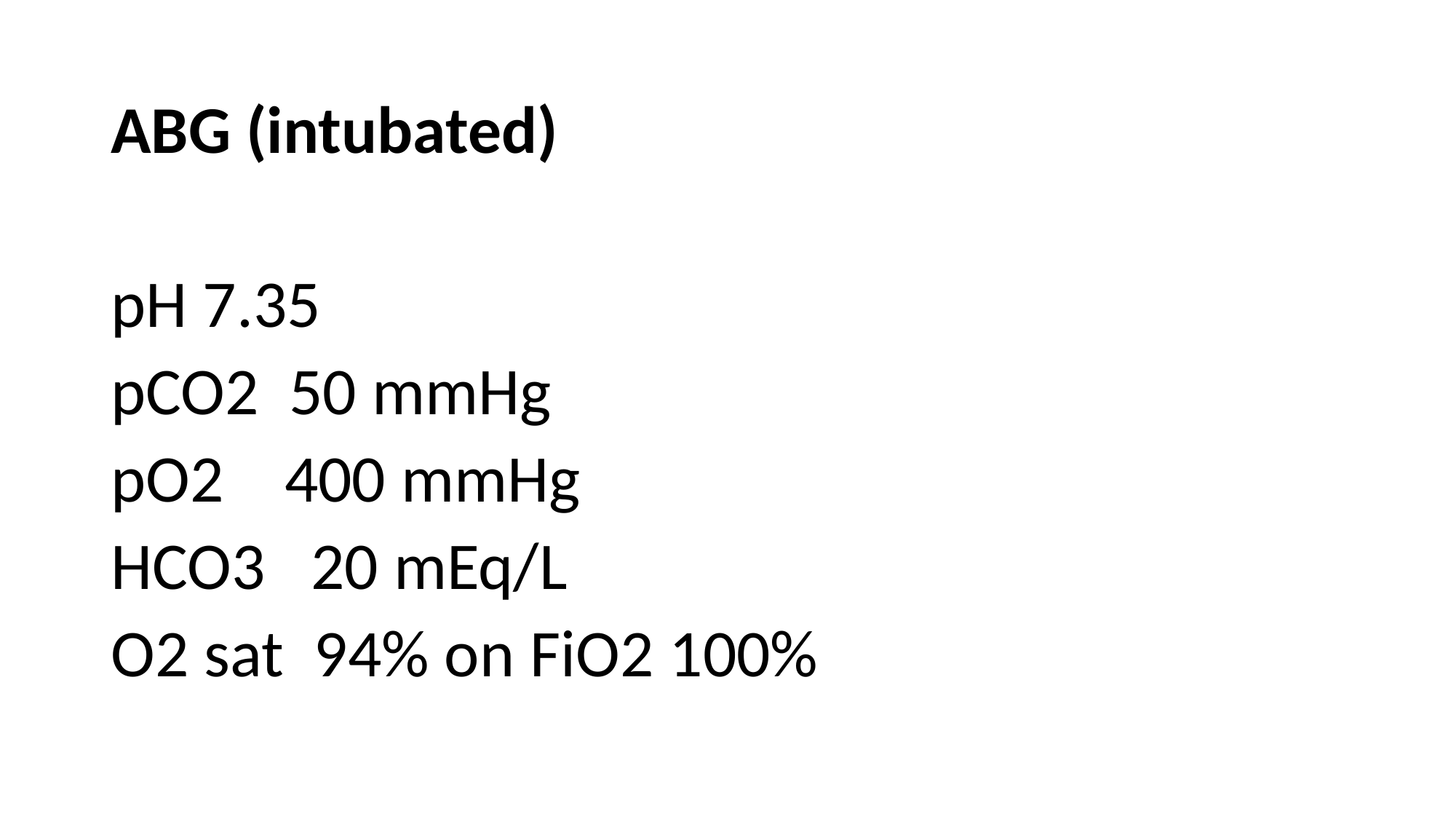

ABG (intubated)
pH 7.35
pCO2 50 mmHg
pO2 400 mmHg
HCO3 20 mEq/L
O2 sat 94% on FiO2 100%

## Slide 15
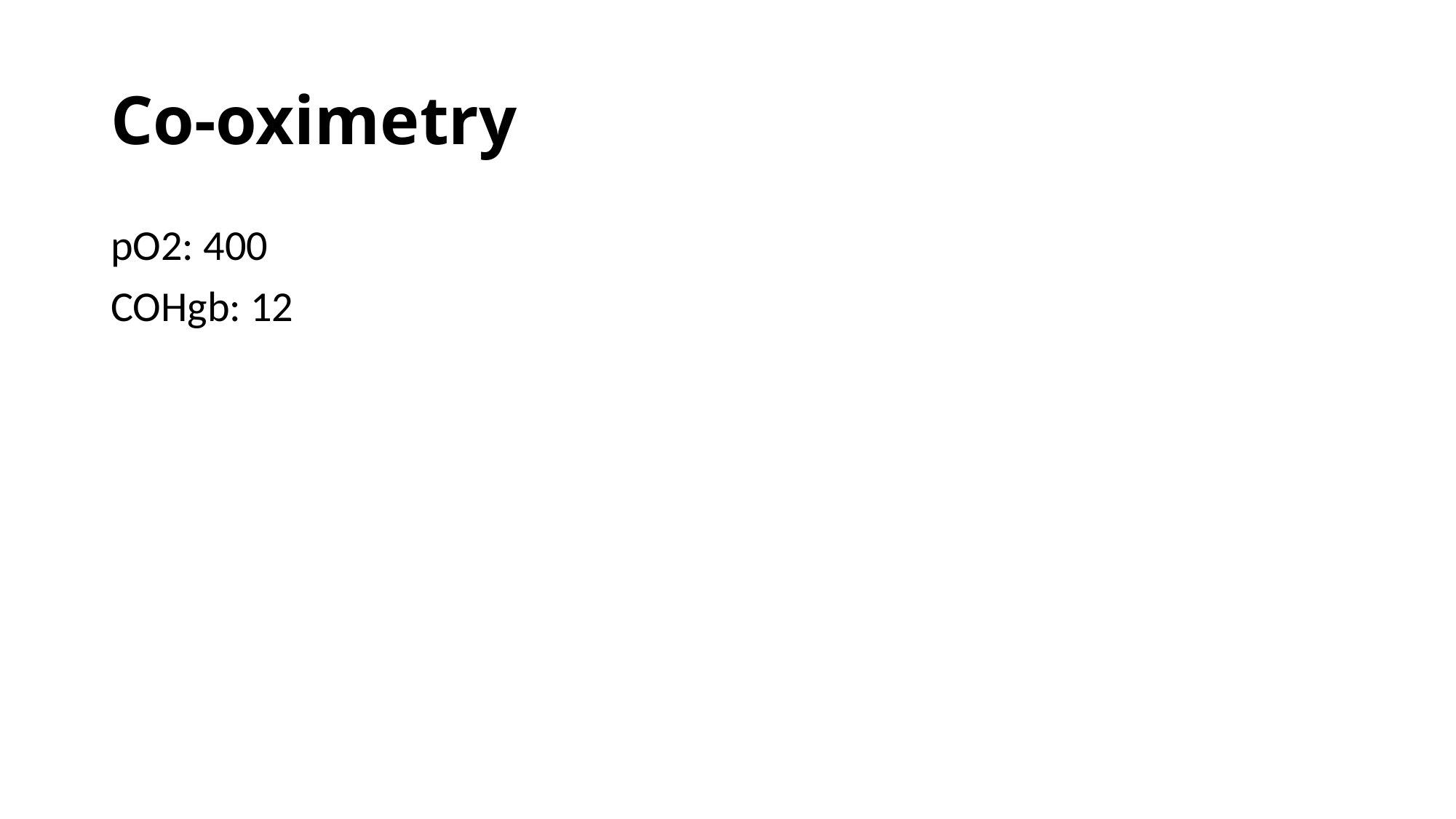

# Co-oximetry
pO2: 400
COHgb: 12
